# Education differences in women’s body weight trajectories: The role of motherhood

**DOI:** 10.1371/journal.pone.0236487

**Published:** 2020-09-21

**Authors:** Hannes Kröger, Liliya Leopold

**Affiliations:** 1 German Socio-Economic Panel, German Institute for Economic Research (DIW), Berlin, Germany; 2 Department of Sociology, University of Amsterdam, Amsterdam, The Netherlands; 3 Professorship of Demography, University of Bamberg, Bamberg, Germany; Anglia Ruskin University, UNITED KINGDOM

## Abstract

Studies have found that education differences in women’s body weight increase until middle adulthood. The explanatory mechanisms behind this increase are not well-understood. This study examined the role of education differences in the *prevalence of motherhood* as a risk factor for weight gain and in *vulnerability* to its effects on weight gain. We used longitudinal data from the German Socio-economic Panel Study. Our sample included 2,668 women aged between 17 and 45 and observed at least twice between 2002 and 2016 (*n* = 13,899 panel observations). We used OLS regression models to estimate initial education differences in body weight and fixed-effects panel regression models to estimate education differences in body-weight trajectories. Motherhood was associated with increasing body weight, and the effects of motherhood on weight gain varied by education. Motherhood partially accounted for the increase of education differences during reproductive age. Until the age of 30, differences in the *prevalence of motherhood* accounted for about 20% of the bodyweight gap between lower and higher educated women. From age 35 until 45, differential *vulnerability* to the effects of motherhood on body weight explained about 15% of the education gap in body weight.

## Introduction

Education differences in women’s body weight are well-documented: Lower educated women are more often overweight and obese than higher educated women in all modern societies [1]. Less is known about why this is the case. It is important to examine the sources of these differences, as weight gains early in life have been found to increase the risk of depression, morbidity, and mortality later in life [2–4] Moreover, overweight and obese people–particularly women–are often socially stigmatized and may be discriminated on the labor market, which in turn negatively affects their psychological and economic well-being [5,6]. As overweight and obesity are most prevalent in lower educated women, they may further intensify social, economic, and health-related disadvantages related to their lower socioeconomic position.

Life-course research has indicated that education differences in body weight develop during early and middle adulthood, usually between ages 20 and 45. During this period, lower educated women gain more weight than higher educated women. After this period, education differences in body weight stabilize [1,7,8]. Importantly, this life-course pattern appears to be specific to women, as education disparities in men’s body weight were found to increase less and at later life stages [1].

These differences suggest that pregnancy and motherhood could explain why lower educated women gain more weight than higher educated women during younger adulthood: These experiences are (a) specific to women, (b) stratified by education, (c) experienced in early to middle adulthood, and (d) constitute a risk factor for weight gain [9]. Lower and higher educated women differ not only in the *prevalence* of this risk factor, but also in their *vulnerability* to its effects on weight gain. Higher educated women have fewer children, are more likely to remain childless [10], and become mothers at older ages [11]. Moreover, higher educated women gain less weight during pregnancy and are more likely to return to their pre-pregnancy weight after giving birth [12]. Taken together, these findings suggest that motherhood and related life changes constitute important mechanisms that may account for the increase of education differences in women’s body weight across early and middle adulthood

Although the idea that motherhood is a potentially influential factor has been articulated in some previous studies [7] it has not been sufficiently addressed empirically. Specifically, previous research mainly focused on identifying education differences in the effects of motherhood on weight gain [12–15], but did not examine the extent to which motherhood explained the life-course increase of education differences in body weight. The present study addressed this gap of knowledge. We examined the role of (1) differential *prevalence of motherhood* as a risk factor for weight gain and (2) the role of differential *vulnerability* to the effects of motherhood on weight gain.

We used longitudinal data from the German Socio-Economic Panel Study (SOEP), spanning the period from 2002 until 2016. These data offer biannual measurements of body weight as well as comprehensive information about socioeconomic indicators and demographic experiences, including pregnancy and childbirth. To examine education differences in women’s body weight trajectories, we selected a sample of 2,668 women comprising 13,899 panel observations and estimated panel models tracing body weight, and education differences therein, from the age of 17 until the age of 45.

## Theoretical background

### Education differences in women’s body weight

Empirical evidence from the past four decades of research has shown that body weight is strongly and negatively associated with socioeconomic status in developed societies [14,16]. A consistent finding is that this association is stronger among women [1]. For example, findings from the U.S., France, and Germany showed that the body weight gap between the top and the bottom tiers of education was three times higher among middle-aged women than among middle-aged men [17].

Studies based on longitudinal and repeated cross-sectional data have shown that education gaps in body weight emerged in early adulthood (approximately during the age between 20 and 30) and still widened until middle adulthood (approximately until the age of 50) [7,8,17,18]. For example, Ailshire and House [7] examined panel data from the Americans’ Changing Lives Survey (ACLS) and found that differences in body weight between lower educated black women and higher educated white women grew especially between the ages of 25 and 54 (by 0.1 of Body Mass Index (BMI) points annually).

Similar tendencies were found in other countries. Molarius and colleagues [17] used repeated cross-sectional data from the comparative “Monitoring Trends and Determinants in Cardiovascular Disease” project (MONICA) of the World Health Organization. Their results revealed that in most developed countries, education differences in BMI grew especially among women below the age of 44. The average increase across ten years ranged between +0.1 BMI points in Italy and Belgium and +1.3 BMI points in Germany and Poland [17]. This evidence suggests that education differences in body weight among women typically increase during a life stage ranging approximately from the early twenties to the mid-forties.

The magnitude of divergence is alarming, given that overweight and obesity are associated with a range of negative health-related, social, psychological, and economic outcomes [2,3]. Earlier onset of overweight and obesity among lower educated women, might mean longer duration of exposure and more severe consequences in terms of physical and mental health [4]. Moreover, overweight and obese people (and especially women) experience disadvantages on the labor market [6], which may be most consequential in early career stages. Finally, even women who are not overweight show lower subjective well-being, higher risk of depression, and lower self-esteem when they gain weight [19]. Understanding the mechanisms behind education differences in weight gain is therefore relevant to the study of inequality in various domains of life.

### Education differences in women’s weight trajectories: The role of motherhood

What explains diverging weight trajectories between education groups throughout early and middle adulthood? Although the phenomenon is well documented, little is known about the underlying mechanisms [8,17,18]. According to the life-course perspective [20], events and transitions experienced early in life represent turning points that channel individuals into adverse trajectories (“chains of risks”) or beneficial trajectories (“protective chains”). A general expectation of the cumulative (dis)advantage theory [21–23] is that the incidence and timing as well as the direction and magnitude of the effects of major events and transitions are socially stratified. As a result, groups that initially lack socioeconomic resources are channeled into adverse trajectories, whereas advantaged groups enter beneficial trajectories, leading to an increase of social inequality over the life course [3,24,25].

Motherhood may represent a turning point for education differences in body weight over the life course. This would imply that motherhood (a) is related to weight gain, (b) differs by education in terms of *prevalence and/or timing*, and/or (c) differs by education in terms of *vulnerability* to its effects on body weight. As we explain in the following sections, previous research has offered empirical evidence in support of each of these arguments.

### Motherhood as a risk factor for weight gain

#### The direct effect of pregnancy

The first pregnancy and each subsequent pregnancy affect women’s weight directly through biological changes. First, glucose alterations during and after pregnancy decrease endogenous estrogens due to fewer ovulatory cycles. This partially accounts for an increased amount of body fat during pregnancy and its persistence after childbirth [26,27]. Second, due to hormonal changes and physical constraints during and after pregnancy, women typically increase their overall calorie intake and reduce their physical activity. According to population studies from the U.S., Sweden, the U.K., and the Netherlands, the direct effect of pregnancy on body weight two years post-partum ranges between 0.6 and 6.5 kg, with an average of 2 kg [28].

#### The chain of risks for weight gain after the transition to motherhood

The effects of motherhood on body weight are not limited to the direct effect of pregnancy. Umberson and colleagues [29] emphasize that motherhood transforms social contexts in a variety of ways that may influence weight gain in the long term. The initial transition to motherhood and subsequent childbearing may generate a chain reaction for further weight gains due to heightened time constraints, financial constraints, and stress levels, which may result in declines in physical activity and increases in calorific intake.

Because women take most of the responsibilities for childcare and parenting, they are also more vulnerable to these mechanisms. In line with these considerations, motherhood has been found to involve greater weight gain and increased obesity risk not only immediately after this transition, but also throughout subsequent life stages until the end of reproductive age and beyond. As a result, the differential weight gain of mothers compared to non-mothers amounted to approximately 5.5 kg [29].

#### Life-course characteristics of motherhood

Both the direct effects of pregnancy and the long-term effects of motherhood on weight gain are moderated by two life-course factors [29]. First, the timing of the initial transition shapes the effect of motherhood on weight trajectories. The direct weight gains after childbirth were found to be stronger for women who gave birth between the ages of 20 and 30, compared to those who became mothers after the age of 30 [28]. Regarding the long-term effects of motherhood on body weight, the earlier a woman becomes a mother, the earlier the onset of the chain of risks, and the longer the exposure to these risks as their effects on weight unfold [1].

Second, parity was also shown to affect weight gain, as weight-related risks associated with pregnancy and with taking care of young children accumulate with the number of children. Mothers who have not returned to their pre-pregnancy weight before their next pregnancy may experience additional weight gains [30]. Moreover, because the typical spacing of births is three years, women who enter higher parities often have to care for more than one young child at the same time. This adds to the effect of early childcare on weight. Longitudinal research has shown that parity strongly contributes to weight gain. Mothers with three or more children had double the risk of obesity compared to those with only one child [31].

### Education, motherhood and weight gain in the German context

If motherhood is a turning point that triggers the increase of education differences in women’s body weight, its prevalence, timing, and/or the vulnerability to its effects on body weight are expected to differ between education groups. Previous research provides evidence in support of each of these arguments in the German context of this study.

First, the prevalence of motherhood differs between education groups in Germany [32,33]. Lower educated German mothers had 2.5 children on average and 27% had three or more children; higher educated mothers had 1.9 children on average and only 12% had three or more children [34].

Second, education also structures the timing of motherhood in Germany, as most women follow the “sequencing norm” of finishing education before having children [35,36]. Among higher educated German women born between 1952 and 1972, the median age at first birth was 33; among lower educated women of the same cohorts, the median age at first birth was 26 [11].

Third, vulnerability to the effects of motherhood on body weight is likely to differ between education groups. Although no study has examined education differences in the effects of motherhood on body weight in Germany, recent research from other Western countries has shown that lower educated women are at a higher risk of excessive weight gain during pregnancy and retain more weight post-partum than higher educated women [12,13,37–39]. For example, in the U.K., lower educated women retained 3.2 kg eight months post-partum, compared to only 1.8 kg among higher educated women [38].

Moreover, the proposed mechanisms behind differential vulnerability to the effects of motherhood on body weight are also likely to operate in the German context. First, the direct effects of pregnancy and childbirth were found to be stronger for women with menarche at younger ages (<12 year old) and for women who gave birth between the age of 20 and 30 (compared to women who became mothers after the age of 30). Although these findings were not linked to education differences in the effects of motherhood, other studies have found that lower educated women experienced their menarche at younger ages [5] and also gave birth at younger ages [11].

Second, compared to higher educated mothers, lower educated mothers more often lack social, economic, and personal resources that help cope with parenting stress [40,41], which in turn may strengthen the chain of risk factors resulting in weight gain. In Germany, protective resources are strongly stratified by education [42,43], suggesting that lower educated women are more vulnerable to the effects of motherhood on weight gain.

Taken together, these considerations and the supporting empirical evidence suggest that motherhood may constitute a turning point in the life-course trajectory of education differences in body weight. Although this idea has been articulated in previous studies [7,44], no research has examined the extent to which the increase of education differences in body weight during reproductive age is explained by differences in the prevalence, timing, and/or effects of motherhood. Previous studies aiming to explain education differences in body weight did not examine the role of these factors from a life-course perspective, particularly in relation to the distinctive pattern of increasing gaps from early to middle adulthood [15].

### Hypotheses

Based on these theoretical considerations and previous evidence, we formulate the following set of hypotheses to guide our empirical analyses: Education differences in women’s body weight emerge and increase during reproductive age (*Hypothesis 1*). Transitions to motherhood and higher parities are associated with an increase in body weight (*Hypothesis 2*). Motherhood is associated with larger weight gains for lower educated women than for higher educated women (*Hypothesis 3*). Education differences in trajectories of body weight are explained by education differences in terms of (a) the prevalence and timing of motherhood, and (b) vulnerability to the effects of motherhood on weight gain (*Hypothesis 4*).

## Data and methods

### Sample selection

Our analyses were based on data from the German Socio-Economic Panel Study [45] v.34. The SOEP is a representative household survey with annual interviews; it started in 1984. Currently it includes data on more than 20,000 respondents from more than 10,000 households drawn from the original sample as well as from regular refreshment samples [46]. The SOEP is among the largest and longest-running representative panel surveys that exist and is widely recognized for maintaining the highest standards of data quality and research ethics. Information on respondents’ body weight and height has been collected in the SOEP since 2002 in biannual intervals. Our analysis drew on these data from an observation period between 2002 and 2016.

In 2002, the anchor year of our study, the sample comprised 29,101 individuals. For the purposes of our investigation, we excluded men (*n* = 14,244) and respondents outside the age range between 17 and 45, as well as those who were older than 35 at first observation (*n* = 11,397). These restrictions centered the analysis on women’s reproductive period and allowed us to examine changes over this life stage. We also excluded immigrant women who were older than 15 years when they arrived in Germany (*n* = 206) to ensure that all women in our sample completed their education in Germany.

Moreover, we excluded observations with extreme values of BMI (lower than 16 or higher than 59) to minimize outlier effects (*n* = 4) and we excluded all observations with missing data on one of the covariates (*n* = 199). Finally, we excluded individuals (*n* = 384) who were observed only once, as our statistical models required at least two observations per respondent. The final sample consisted of 2,668 women, comprising 13,899 panel observations. All sample exclusions are summarized in Table A1 in the S1 Appendix.

### Measure of education

Education was measured as a time-constant indicator of the *highest degree attained until the end of the observation window*. Our observation window of up to 14 years covered the age span during which even the youngest respondents have most commonly received their highest educational degrees, ensuring that our measure accurately reflected education level.

Our education measure was based on the “Comparative Analysis of Social Mobility in Industrial Nations” classification (CASMIN). We grouped the CASMIN categories as follows: The bottom category comprised individuals holding lower secondary degrees with completed vocational qualification or less (CASMIN 1a–1c); intermediate education ranged from intermediate secondary degrees to higher secondary degrees with vocational qualification (CASMIN 2a–2c); the top category included respondents holding tertiary degrees (CASMIN 3a–3b). In our sample, 24.2% of women were lower educated, 54.2% had intermediate degrees, and 21.6% were higher educated (see Table 1). The share of missing information on education amounted to only 2.9%. Cases with missing information on education were removed from the analyses (see Table A1 in the S1 Appendix). In substantive terms, these categories represent meaningful comparison groups for the study of the role of motherhood in education differences in body weight, as the prevalence and the timing of motherhood–as well as resources that may help to prevent weight gain due to motherhood–differ substantially between these groups.

**Table 1:** Descriptive statistics.

	*Total*					*Lower education*				*Intermediate education*				*Higher education*				*Difference*	
	M	SD	Min	Max	Miss	M	SD	Min	Max	M	SD	Min	Max	M	SD	Min	Max	L-H^a^	I-H^b^
Body weight (kg)	67.07	13.61	37.00	155.00	1.06	69.51	15.61	37.00	155.00	67.53	13.88	41.00	145.00	64.18	10.47	44.00	120	5.33***	3.35***
Height (cm)	167.58	6.43	140.00	194.00	0.03	165.74	6.61	140.00	194.00	167.83	6.36	148.00	189.00	168.56	6.11	150.00	187	-2.82***	-0.73*
BMI (kg/m^2^)	23.88	4.67	16.04	54.92	1.11	25.28	5.41	16.20	54.92	23.96	4.71	16.04	52.08	22.58	3.44	16.14	42.52	2.71***	1.39***
Birth last two years	0.09	0.29	0.00	1.00	0.00	0.08	0.28	0.00	1.00	0.09	0.29	0.00	1.00	0.10	0.30	0.00	1.00	-0.02**	-0.012
Number of children:	ref.					ref.				ref.				ref.					
No children	0.44	0.50	0.00	1.00	0.00	0.31	0.46	0.00	1.00	0.42	0.49	0.00	1.00	0.59	0.49	0.00	1.00	-0.28**	-0.18**
1 child	0.23	0.42	0.00	1.00	0.00	0.23	0.42	0.00	1.00	0.25	0.43	0.00	1.00	0.18	0.38	0.00	1.00	0.05**	0.07***
2 children	0.24	0.43	0.00	1.00	0.00	0.27	0.44	0.00	1.00	0.26	0.44	0.00	1.00	0.19	0.39	0.00	1.00	0.08***	0.07***
> = 3 children	0.09	0.29	0.00	1.00	0.00	0.19	0.39	0.00	1.00	0.08	0.27	0.00	1.00	0.04	0.21	0.00	1.00	0.15***	0.03***
Age	31.93	6.73	16.50	45.58	0.00	32.04	6.84	16.50	45.33	32.02	6.72	16.50	45.58	31.66	6.67	16.50	45.42	0.388	0.36
Age (centered)	12.93	6.73	-2.50	26.58	0.00	13.04	6.84	-2.50	26.33	13.02	6.72	-2.50	26.58	12.66	6.67	-2.50	26.42	0.38	0.36
Age at 1^st^ birth	26.98	5.02	15.00	44.00	0.00	23.83	4.47	16.00	44.00	26.92	4.51	15.00	41.00	30.23	4.61	17.00	41.00	-6.40***	-3.31**
Age at 2^nd^ birth	29.74	4.70	18.00	43.00	0.00	26.79	4.30	18.00	41.00	29.95	4.27	20.00	43.00	32.53	4.25	21.00	43.00	-5.74***	-2.58**
Age at 3^rd^ birth	31.15	4.72	20.00	44.00	0.00	29.23	4.78	20.00	42.00	31.49	3.98	22.00	41.00	34.39	4.29	22.00	44.00	-5.16***	-2.90**
Proportion	100%					22.26%				54.54%				23.20%					
Individuals	2,668					594				1455				619					
Observations	13,899					2905				7392				3602					

Data are from SOEP v.34, own calculations.

^a^ The column L-H indicates the differences between the means of lower educated women and the means of higher educated women.

^b^ M = mean, Miss = Missing (%). The column I-H shows the differences between the means of intermediately educated women and the means of higher educated women. Statistical significance is assessed by the t-tests.

*** indicates p < 0.001

** indicates p < 0.01

* indicates p < 0.05.

**Fig 1:**
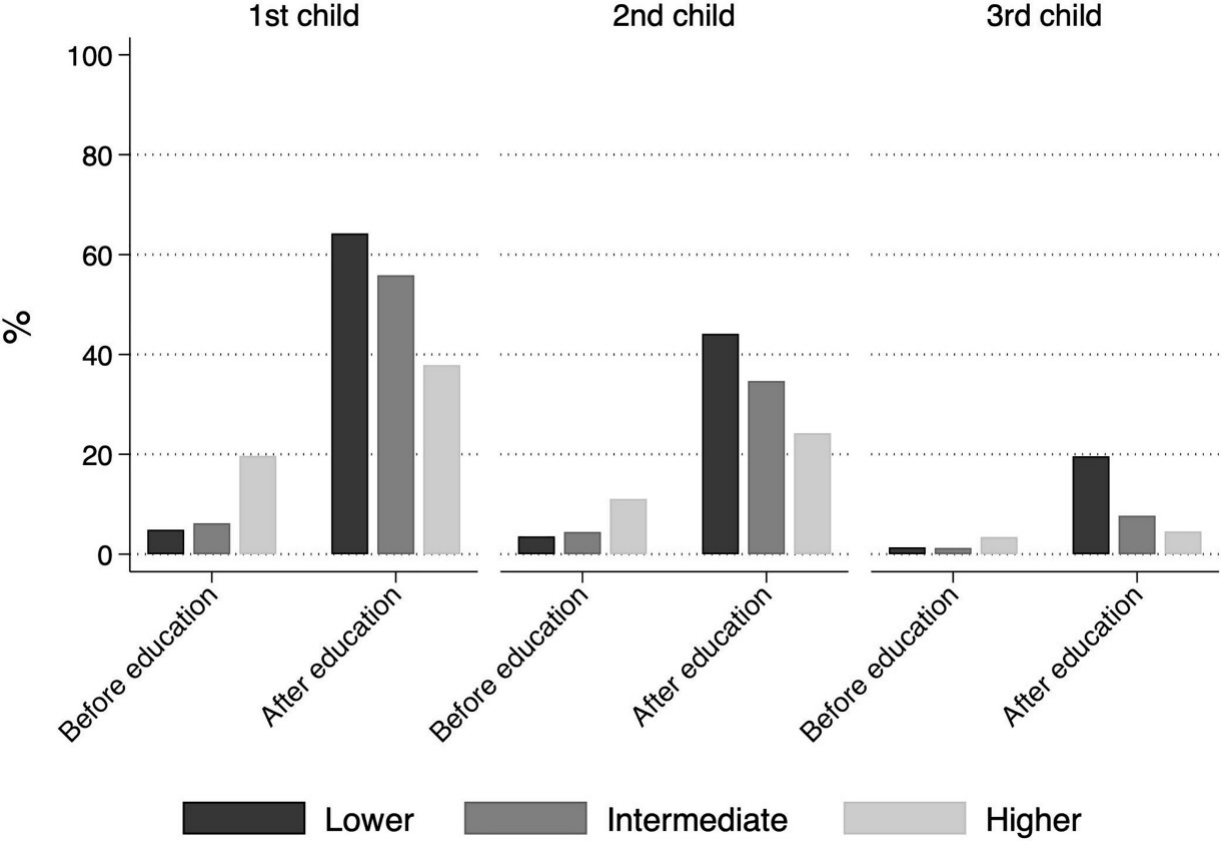
Timing of motherhood by education. Data are from SOEP, v.34; own calculation.

First, the time spent in the education system strongly determines the timing of motherhood in Germany. Due to a strong sequencing norm, most women in Germany become mothers after completing their education (Fig 1) [11]. As visible from Table 1, lower educated mothers’ average age at first birth was 24, approximately 6 years less than higher educated mothers’ average age at first birth.

Second, social and economic resources are strongly stratified by education in Germany, which is mainly due to a selective and rigid school system characterized by early tracking and a strong connection between educational degrees and labor market outcomes.

These conditions favor the reproduction of initial advantages and disadvantages related to social origin, and stratify economic outcomes in later life along educational lines [47,48]. These features of the German context suggest that lower educated women are more vulnerable to the effects of motherhood because they are often deprived of resources protecting them from the chain of risks resulting in weight gain.

### Measures of body weight

Our outcome measure was body weight in kilograms (controlled for self-reported height). This measure was based on self-reported information on respondents’ weight and height, assessed biannually since 2002. Self-reported weight and height measures were shown to be closely related to physical measurements. While people report their height relatively accurately, the discrepancy between measured and self-reported weight is larger and related to age, education, sex, and body weight itself. Specifically, younger, higher-educated, and rather heavy women tend to underestimate their weight [49–51]. However, the size of these discrepancies is relatively small as about 85% of self-reports of weight correspond closely with measured weight [52]. For these reasons, self-reported weight and height are considered valid measures in studies of population health, although some caution is warranted with the interpretation of effect sizes. In our models, we focus on body weight (controlled for self-reported height) to ease the interpretation of effect sizes. As BMI provides important additional information regarding the thresholds of overweight and/or obesity we also presents results for BMI. Table 1 provides descriptive information on how body weight and height varied between education groups. Averaged across all panel observations, lower educated women were smaller but 2kg heavier than women with intermediate degrees and 5kg heavier than women with higher degrees.

### Measures of motherhood

In studies on body weight, motherhood is typically measured by indicators for the number of children [9,31]. In our analysis, we complemented this standard specification by a measure of short-term effects of motherhood related to pregnancy and the time after birth. The first measure for the number of children was a time-varying categorical variable distinguishing between observations in which women were childless (reference category), had one child, had two children, or had three or more children. The second measure capturing the short-term effects of pregnancy and motherhood on body weight was a dummy variable coded 1 if women gave birth in the year of the interview or in the previous year and 0 otherwise. A number of studies have suggested that the effects of motherhood might be particularly strong at higher parities. To examine this possibility, we additionally assessed (1) whether the short-term effects were larger at higher parities, and (2) whether the short-term effects at higher parities differed by education. The results showed that the short-term effect of motherhood was only slightly larger at parities of 2 and above. It was also slightly larger among lower educated women. Because all substantive conclusions remained the same under this alternative specification, we decided to keep the model parsimonious and did not include further interactions.

Moreover, as being pregnant in the reference year might lead to an underestimation of the effects of motherhood, we assessed whether our results were robust to excluding observations during pregnancy. This exclusion did not influence on our results. For the main analyses, we kept these observations in the data. Table 1 shows large education differences in the motherhood indicators. Lower educated women were childless in only 32% of the observations, compared to 42% for intermediately educated women, and 61% for higher educated women. Large education differences were also observed for higher parities. Lower educated women had three or more children in 19% of their observations, intermediately educated women in 8% of their observations, and higher educated women in 4% of their observations. These descriptive results were in line with previous research [34] and our theoretical expectations about education differences in motherhood.

**Fig 2:**
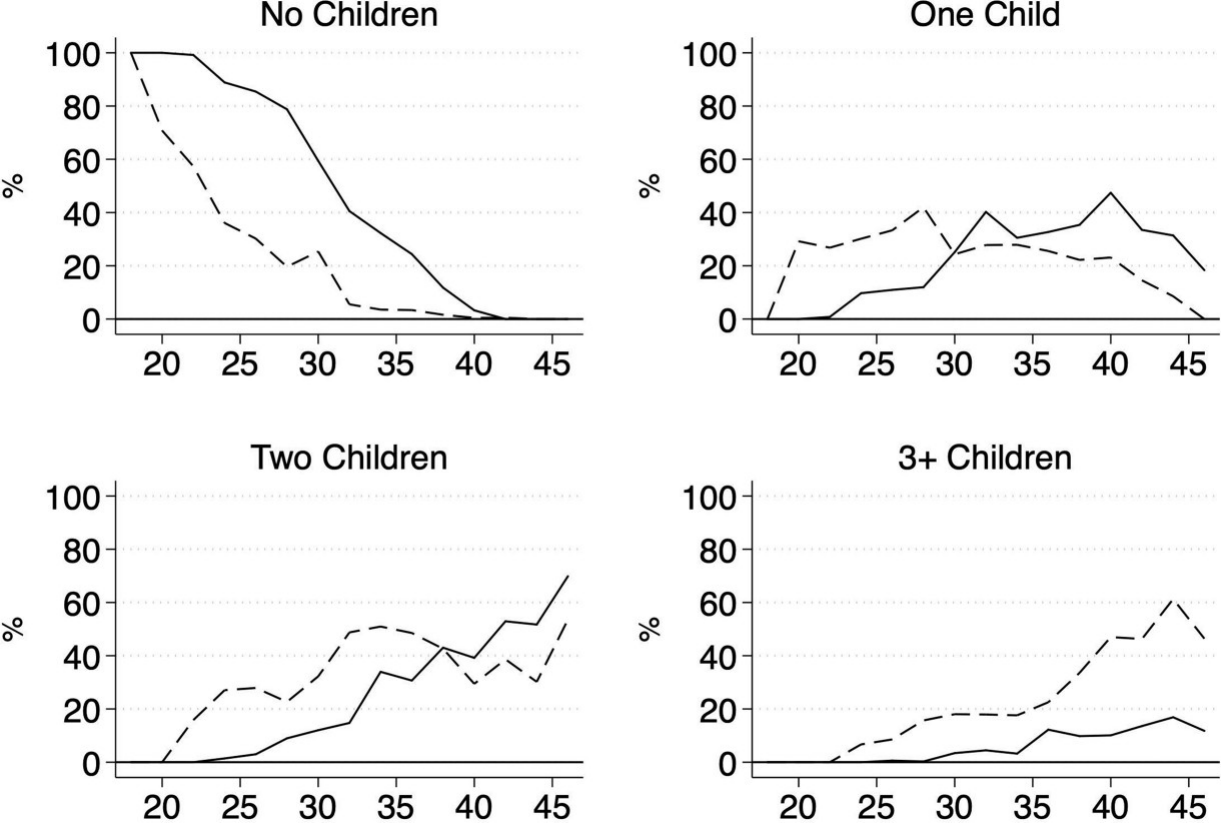
Distribution of the number of children categories by education and age. Data are from SOEP, v.34; own calculations; Lower education = dashed lines; Higher education = solid lines.

Fig 2 further shows that lower educated women became mothers earlier in life than higher educated women. Higher educated women had ‘caught up’ on having one child (by the age of 30) or two children (by the age of 38), but not on having at least three children. This category was much more prevalent among lower educated women across the entire age range of our study.

### Modelling of age effects

Age was measured in years and months at the time of the interview. The age range covered was 16.5 to 45.6 years, with a mean of 31.6 years. In the models (Table 2), we included linear and squared terms of age, centered at the age of 30.

**Table 2:** Panel models for body weight.

	M1- Baseline	M2—Prevalence	M3—Effects
	Coef [CI]	Coef [CI]	Coef [CI]
Age	0.85***	0.75***	0.69***
	[0.44,1.26]	[0.39,1.12]	[0.36,1.02]
Age^2^	-0.01	-0.00	-0.00
	[-0.02,0.00]	[-0.02,0.01]	[-0.01,0.01]
Age * Education			
Intermediate*Age	0.03	0.01	0.06
	[-0.41,0.47]	[-0.40,0.42]	[-0.30,0.43]
Higher*Age	-0.27	-0.29	-0.20
	[-0.71,0.16]	[-0.69,0.11]	[-0.56,0.16]
Age^2^ * Education			
Intermediate*Age^2^	-0.01	-0.01	-0.01
	[-0.02,0.01]	[-0.02,0.01]	[-0.02,0.00]
Higher*Age^2^	0.00	0.00	-0.00
	[-0.01,0.01]	[-0.01,0.01]	[-0.01,0.01]
Number of children			
No children		Ref.	Ref.
1 child		0.98*	0.51
		[0.18,1.78]	[-2.04,3.05]
2 children		1.35*	3.22
		[0.13,2.58]	[-1.60,8.04]
> = 3 children		1.98	2.72
		[-0.09,4.04]	[-3.44,8.89]
Birth this or last year		2.37***	2.74***
		[1.77,2.97]	[1.16,4.33]
Motherhood * Education			
1 Child*Intermediate			0.96
			[-1.82,3.75]
1 child*Higher			0.23
			[-2.53,2.98]
2 children *Intermediate			-1.88
			[-6.92,3.15]
2 children *Higher			-2.75
			[-7.79,2.29]
> = 3 children *Intermediate			-0.07
			[-6.75,6.61]
> = 3 children *Higher			-1.51
			[-8.16,5.14]
Birth *Intermediate			-0.47
			[-2.23,1.29]
Birth *Higher			-0.62
			[-2.47,1.22]
Education baseline weight (Eq 1B)			
Low	62.27***	62.27***	62.27***
	[60.43,64.10]	[60.43,64.10]	[60.43,64.10]
Intermediate	61.11***	61.11***	61.11***
	[59.80,62.43]	[59.80,62.43]	[59.80,62.43]
Higher	58.94***	58.94***	58.94***
	[57.54,60.34]	[57.54,60.34]	[57.54,60.34]
F-statistic compared to Model 1		28.59 (4,2667)	11.59 (12,2667)
p-value		< 0.00	< 0.00
Observations	13899	13899	13899
Individuals	2668	2668	2668

Data are from SOEP v.34; own calculations; age and age squared are centered at the values corresponding to the age of 19; height centered at the mean of 167 cm; *γ*_1*i*_ is the random slope; *u*_1*i*_ is the random intercept; 95%-CI in brackets.

*** p < 0.001

** p < 0.01

* p < 0.05

**Fig 3:**
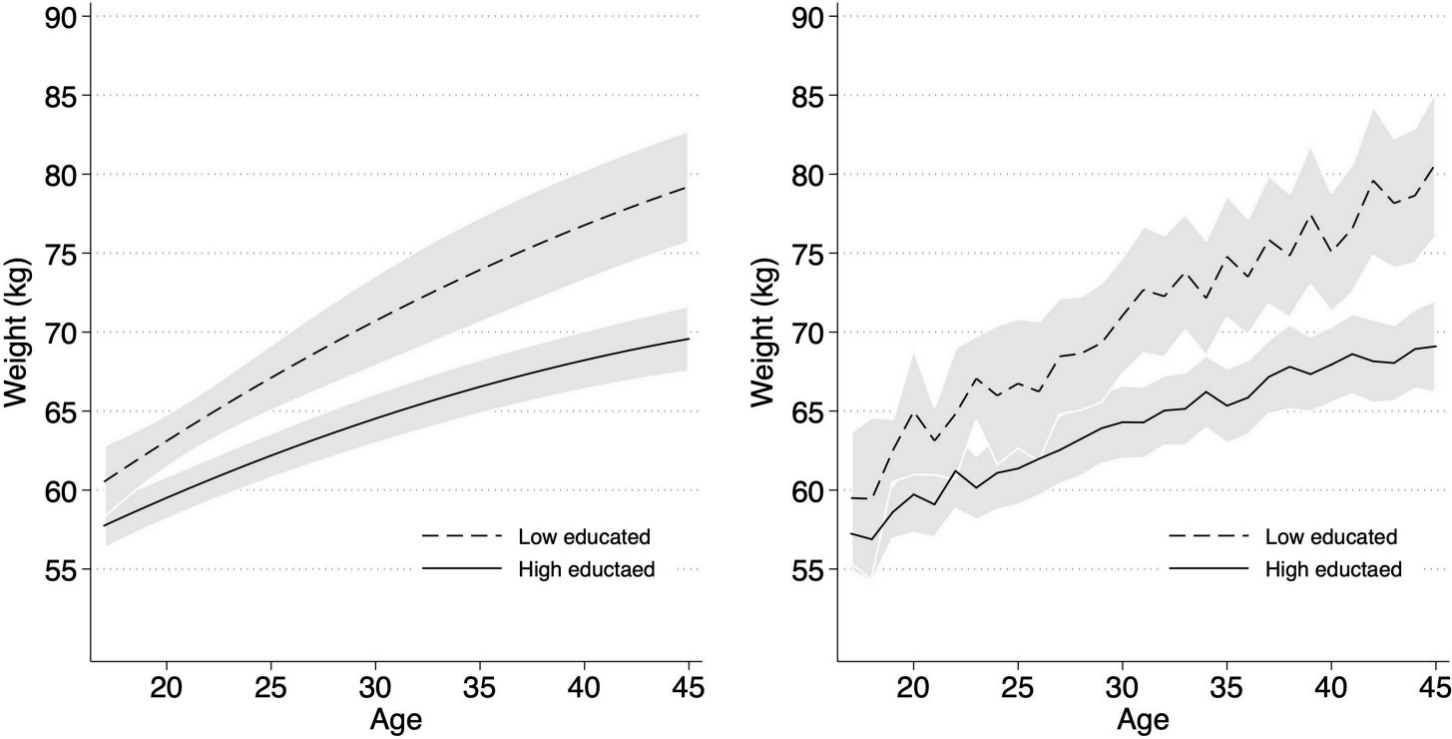
Predicted body weight trajectories form the preferred parametric model specification and from a semi-parametric model specification. Data are from SOEP, v.34; own calculations; (a) predicted body-weight trajectories form the preferred parametric model specification (model M1, Table 2); (b) predicted body-weight trajectories form a semi-parametric model specification; 95% CI = grey areas.

We tested for alternative specifications of the functional form of age effects, such as linear, squared, and cubic terms of age as well as the most flexible non-parametric specification with dummies for each year of age. According to the Bayesian Information Criterion (BIC) and diagnostic plots, a model that included linear and squared terms of age and their interactions with education provided the best fit to the data. Fig 3 shows a comparison between our preferred model for body weight and a semi-parametric model estimating body weight differences separately for each age. The figure indicates that our parametric model accurately described body weight trajectories for different educational groups.

Given the structure of our data, in which individuals enter the data at different ages and are observed for a maximum of 15 years, cohort effects might be confounded with age patterns. To assess the influence of cohort effects, we examined interactions between cohort and age (linear, squared, cubic) and education. According to the BIC criterion and diagnostic plots, these additional interactions worsened the model fit. Therefore, we did not control for cohort in our analyses. This means that our analysis assessed the relationship between education, age, and body weight averaged over all cohorts.

### Weighting and attrition

We used cross-sectional survey weights to ensure that our data were representative of the German population. These weights correct for oversamples included in the SOEP as well as systematic panel attrition before our starting year of 2002. The survey weights were calculated by the SOEP research team [53]. After weighting, our sample was representative for women aged 17 to 45 living in German households in 2002 [54].

Furthermore, we analyzed whether panel dropout was related to our variables of interest, estimating a discrete-time survival model for the probability of dropout, defined as providing no or no valid information before the end of the observation period in 2016. Our results indicated that dropout was barely related to motherhood, education, and body weight. Mothers were slightly more likely to stay in the panel and this did not vary by education or body weight. Higher educated women were slightly more likely to drop out when they gained weight, while lower educated women were slightly more likely to drop out when they lost weight. Although this suggested that educational differences in body weight might be overestimated if selective dropout is ignored, this bias is very small.

### Analytic strategy

The aim of our analyses was to estimate age-related change of education differences in body weight and to assess the role of motherhood in this process.

To estimate the effects of motherhood, we used fixed-effects models, which account for time-stable characteristics of women that may confound the effect of motherhood. The fixed-effects estimator draws only on within-person change over time, controlling for all time-constant characteristics of women, regardless of whether they are measured or not.

In our case this means that the effects of motherhood are estimated solely from actual transitions to motherhood and to higher parities. Body weight is observed before pregnancy and changes are observed during pregnancy and after giving birth. The main advantage of this approach is that time-constant confounders are rendered inconsequential because the estimates are based solely on change within persons over time.

To estimate initial educational differences in body weight, we used a between-person estimator, given that a fixed-effects model does not estimate the effects of a time-constant variable such as women’s education. To obtain a between-person estimator, we used OLS to calculate initial educational differences in body weight at age 19.

This means that our models combined within-person fixed-effects estimators with an initial between-person OLS estimator [55]. This approach is similar to the hybrid model [56,57] and provides within-person fixed-effects estimates for the effects of age and motherhood (i.e., within-effects) while allowing for the inclusion of time-constant variables such as education and height to estimate initial differences in body weight (i.e., between-effects).

Based on this approach, we estimated three models to answer the main questions of our study. The first model described the phenomenon we sought to explain, namely education differences in body weight across the reproductive period of women’s life courses (Hypothesis 1). The second model assessed the relationship between motherhood and body weight (Hypothesis 2). The third model examined education differences in the effects of motherhood and the extent to which motherhood, and differences in its effects, explained the life-course pattern of education differences in body weight (Hypothesis 3).

We specified our first model as follows:

$$Y_{it}=\alpha_{t0}+\left(\gamma_{1}+\sum_{k\in \{int,high\}}\gamma_{3,k}{s_{k}}_{i}\right)z_{it}+\left(\gamma_{2}+\sum_{k\in \{int,high\}}\gamma_{4,k}{s_{k}}_{i}\right)z_{it}^{2}+\sum_{k\in \{int,high\}}\delta_{k}{s_{k}}_{i}+u_{i}+\epsilon_{it}$$ (M1)

where *Y*_*it*_ is the outcome (body weight) for an individual *i* at time point *t*. *γ*_1_ and *γ*_2_ are the parameters of linear (*z*_*it*_) and squared ($z_{it}^{2}$) terms of age defined for the reference group of lower educated women (k = 0). *γ*_3,*k*_ and *γ*_4,*k*_ are the differences in linear and quadratic body weight trajectories between the lower, intermediate, and higher educated women. ${s_{k}}_{i}$ is a time-constant indicator of education. *u*_*i*_ represents the individual-specific time-constant intercept (including all time constant confounders). *ϵ*_*it*_ is the idiosyncratic observation-specific error term with an expectation of zero and assumed to be uncorrelated with all other terms in the model. ∑_*k*∈{*int*,*high*}_ *δ*_*k*_ indicates initial differences in body weight between education groups.

The fixed-effects estimator used to estimate the effect of age on change in body weight was specified as follows:

$$Y_{it}-{\bar{Y}}_{i}=\left({\hat{\gamma }}_{1}+\sum_{k\in \{int,high\}}{\hat{\gamma }}_{3,k}{s_{k}}_{i}\right)(z_{it}-{\bar{z}}_{i})+\left({\hat{\gamma }}_{2}+\sum_{k\in \{int,high\}}{\hat{\gamma }}_{4,k}{s_{k}}_{i}\right)(z_{it}^{2}-{\bar{z}}_{i}^{2})+{\hat{\epsilon }}_{it}$$ (1A)

where $Y_{it}-{\bar{Y}}_{i}$ indicates within-person change in body weight, calculated as the body weight of individual *i* at time point *t* minus the average body weight of an individual *i*, and ($z_{it}-{\bar{z}}_{i}$) and $\left(z_{it}^{2}-{\bar{z}}_{i}^{2}\right)$ represent within-person changes in age and age squared; *u*_*i*_ is eliminated from the model. Although this approach solves the problem of time-constant unobserved confounding, it also eliminates the time-constant average level of weight represented by *α*_*t*0_ and the respective initial differences between the educational groups represented by $\delta_{k}{s_{k}}_{i}$ in Eq 1. Therefore, we added an estimation step for initial educational differences at the age of 19.

$$Y_{i19}={\hat{\alpha }}_{t0}+{\hat{\delta }}_{k}{s_{k}}_{i}+\hat{\beta }H_{i}+{\hat{e}}_{i0}$$ (1B)

For the estimation of the initial differences (represented by *Y*_*i*19_), we additionally controlled for self-reported height ($\hat{\beta }H_{i}$) to account for structural differences in height between education groups. The results of these models are presented in Table 2 (M1) and in Fig 3.

In our second model, we assessed how motherhood was related to body weight. For this, we added both indicators of motherhood (***M***_*it*_)–the number of children and the indicator for whether the respondent gave birth in the year of the interview or in the previous year.

$$Y_{it}={\dot{\alpha }}_{t0}+\left({\dot{\gamma }}_{0}+{\dot{\gamma }}_{1i}+\sum_{k\in \{int,high\}}{\dot{\gamma }}_{3,k}{s_{k}}_{i}\right)z_{it}+\left({\dot{\gamma }}_{2}+\sum_{k\in \{int,high\}}{\dot{\gamma }}_{4,k}{s_{k}}_{i}\right)z_{it}^{2}+\sum_{k\in \{int,high\}}{\dot{\delta }}_{k}{s_{k}}_{i}+M_{it}\zeta +\dot{u_{i}}+{\dot{\epsilon }}_{it}$$ (M2)

The parameters of the model are also estimated using the within-person fixed-effects estimator [55] specified as follows:

$$Y_{it}-{\bar{Y}}_{i}=\left({\hat{\gamma }}_{1}+\sum_{k\in \{int,high\}}{\hat{\gamma }}_{3,k}{s_{k}}_{i}\right)(z_{it}-{\bar{z}}_{i})+\left({\hat{\gamma }}_{2}+\sum_{k\in \{int,high\}}{\hat{\gamma }}_{4,k}{s_{k}}_{i}\right)(z_{it}^{2}-{\bar{z}}_{i}^{2})+(M_{it}-{\bar{M}}_{i})\hat{\zeta }+{\hat{\epsilon }}_{it}$$ (2)

where $(M_{it}-{\bar{M}}_{i}){\hat{\zeta }}_{s}$ represent within-person changes in the motherhood indicators. The results of this model are presented in Table 2 (M2).

In our final model, we further added interactions between education and both indicators of motherhood to assess whether the effects of motherhood on body weight differed between educational groups.

$$Y_{it}=\ddot{\alpha_{t0}}+\left({\ddot{\gamma }}_{0}+{\ddot{\gamma }}_{1i}+\sum_{k\in \{int,high\}}{\ddot{\gamma }}_{3,k}{s_{k}}_{i}\right)z_{it}+\left({\ddot{\gamma }}_{2}+\sum_{k\in \{int,high\}}{\ddot{\gamma }}_{4,k}{s_{k}}_{i}\right)z_{it}^{2}+\sum_{k\in \{int,high\}}{\ddot{\delta }}_{k}{s_{k}}_{i}+M_{it}{\ddot{\zeta }}_{s}+{\ddot{u}}_{i}+{\ddot{\epsilon }}_{it}$$ (M3)

where ${\ddot{\zeta }}_{s}=(\zeta_{low}+\sum_{k\in \{int,high\}}\zeta_{k}{s_{k}}_{i})$. Educational differences in the effects of motherhood on body weight are represented by ${\ddot{\zeta }}_{s}$, which allows the coefficients of the birth variables to vary across educational groups. *ζ*_*low*_ represents the estimated body weight of the reference group of lower educated women. The model is estimated analogously to (2), but allowing for differential effects of motherhood as specified. The results of this model are presented in Table 2 (M3).

Combining the results from models 1b and 2 gives us the model-based predictions for weight trajectories of different educational groups.

$${\hat{Y}}_{it}={\hat{\alpha }}_{t0}+{\hat{\delta }}_{k}{s_{k}}_{i}+\left({\hat{\gamma }}_{1}+\sum_{k\in \{int,high\}}{\hat{\gamma }}_{3,k}{s_{k}}_{i}\right)z_{it}+\left({\hat{\gamma }}_{2}+\sum_{k\in \{int,high\}}{\hat{\gamma }}_{4,k}{s_{k}}_{i}\right)z_{it}^{2}+M_{it}{\hat{\zeta }}_{s}$$ (3)

In order to answer our main research question–to what extent differences in the prevalence and effects of motherhood account for educational differences in trajectories of body weight (Hypothesis 4)–we estimated a counterfactual scenario based on Eq 3. In this scenario, we estimated, first, what the age pattern of change in body weight among lower educated women would look like if they had the same *prevalence* of motherhood as higher educated women. For our sample, this means that we predicted how body weight among lower educated women would change with age if they had no children in 61% instead of 32% of their observations, if they had one or two children in 17% instead of 23% of their observations, if they had at least three children in 4% instead of 19% of their observations, and if they had given birth in 11% instead of 9% of their interview years. Second, we used the estimated coefficients of both indicators of motherhood on the body weight of higher educated women ($\zeta_{low}+\sum_{k\in \{int,high\}}\zeta_{k}{s_{k}}_{i}$) for the calculation of the counterfactually predicted weight trajectory of lower educated women. This allowed us to additionally account for education differences in the *effects* of motherhood on body weight.

Using these two counterfactual assumptions, the implied counterfactual differences in weight (${\ddot{\Delta }}_{z}$) that remain are defined as:

$${\ddot{\Delta }}_{z}={\hat{Y}}_{z|s=low,M={\bar{M}}_{s=high},H_{low}=\bar{H}}-{\hat{Y}}_{z|s=high,M={\bar{M}}_{s=high},H_{high}=\bar{H}}$$ (4)

for the scenario in which lower educated women had the same prevalence of motherhood as higher educated women. For both prevalence and effects, the difference is defined as:

$${\ddot{\Delta }}_{z}={\hat{Y}}_{z|s=low,{\ddot{\zeta }}_{s}=\zeta_{low}+\zeta_{high},M={\bar{M}}_{s=high},H_{low}=\bar{H}}-{\hat{Y}}_{z|s=high,M={\bar{M}}_{s=high},H_{high}=\bar{H}}$$ (5)

As in the equations above, *z* represents age, ***M*** represents the motherhood indicators, and *ζ* represents the association between motherhood and education.

**Fig 4:**
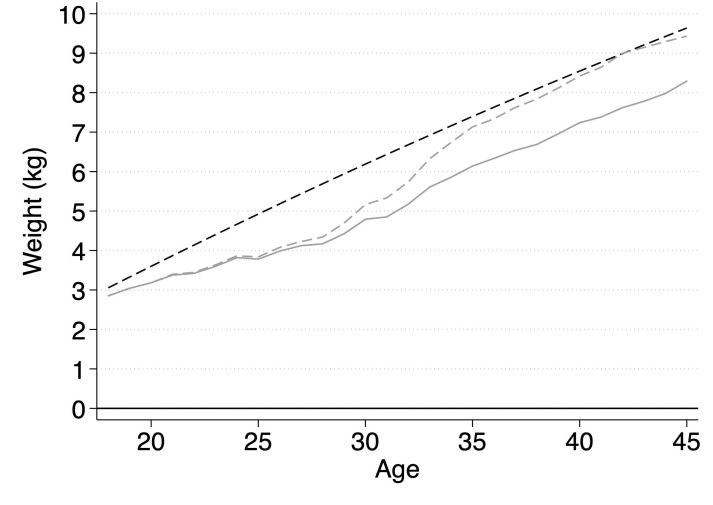
Predicted differences in body weight between lower and higher educated women with and without adjustment for motherhood. SOEP data, v.34; own calculations; height is fixed at the average of 167 cm in both models; black solid reference line = body weight of higher educated women, black dashed line = difference in body weight between lower educated women and higher educated women based on model M1, Table 2; grey dashed line = difference in body weight between lower educated women and higher educated women adjusted for differences in prevalence of motherhood; grey solid line = difference in body weight between lower educated women and higher educated women adjusted for differences in prevalence and effects of motherhood (M3), Table 2.

As educational differences in our outcomes are expected to change with age, it is also likely that the contribution of motherhood–both with respect to prevalence and to effects–to these differences is not uniform across the age range. To account for this, we calculated the contribution of motherhood according to the counterfactual scenario at different ages (*z*). The results of the counterfactual analysis are presented in Fig 4 and in Table 4.

## Results

The first step of our analysis was to estimate the life-course pattern of education differences in women’s body weight during their reproductive period. If motherhood drives education differences in body weight, these differences are expected to increase especially during reproductive age. The results of this first step are shown in Fig 3 and the corresponding model M1 (Table 2).

As indicated by the age effects, the education effects, and their interactions, education differences in body weight were large and increased with age. At the age of 19, higher educated women weighed about 6.5 kg less than lower educated women. As the negative interaction term between linear age and higher education indicates, body weight increased less with age among higher educated women.

Fig 3 visualizes these results. Estimates for higher educated women are represented by solid lines and estimates for lower educated women are represented by dashed lines. The left-hand panel shows the parametric estimates of Model M1; the right-hand panel shows the non-parametric model estimates. The plots demonstrate that our parametric model provided a good fit with non-parametric estimates of education differences in body weight across the age range under study.

As Fig 3 shows, education differences in body weight increased from about 3 kg at age 17 (difference not statistically significant) to gaps of 6.5 kg by the age of 30. Subsequently, the gaps increased further, albeit more slowly, reaching almost 8.5 kg at the age of 40.

The second step of our analysis assessed the effects of motherhood on body weight. For motherhood to explain the education differences found in model M1, it must be related to body weight. Model M2 supports this argument. Compared to having no children, having one child was associated with an increase of about 1 kg, having two children with 1.3 kg, and having at least three children with about 2 kg.

Moreover, independent of the number of children, having given birth in the year of the interview or in the previous year was associated with about 2.7 kg of additional body weight.

In the third step of our analysis, we examined whether the relationship between motherhood and body weight differed between education groups. We expected education groups to differ not only regarding the prevalence and timing of motherhood, but also regarding their vulnerability to the effects of motherhood on weight gain. As the interaction effects in model M3 indicate, higher and lower educated women differed in their weight gains associated with motherhood. Given our limited sample size (for cell sizes, see Table A3 and Table A4 in the S1 Appendix), the interaction effects were not statistically significant. However, the estimates were not only substantial in size and in line with theoretical considerations, but also showed a pattern of results that was similar to other studies analyzing education differences in the effects of motherhood on body weight with much larger data sets [13]. As recommended in recent methodological studies, estimates that are substantial in size albeit not statistically significant should be interpreted substantively–especially if they fit in the general pattern of results and/or are in line with other studies using larger data sets [58,59].

To ease the interpretation, we calculated model-based differences in body weight between higher and lower educated women dependent on their motherhood status. These calculations are presented in Table 3.

**Table 3:** Education differences in effects of motherhood on body weight at age 30.

	*Body weight*							
	Lower educated		Higher educated					
	Predicted weight	Change	Predicted weight	Change	Diff. in predicted weight	p-value	Diff. in change	p-value
Number of children								
No children (reference)	69.74		63.98		5.76	0.00		
1 child	70.25	+0.51	64.71	+0.73	5.54	0.01	-0.22	0.87
2 children	72.96	+3.22	64.44	+0.46	8.52	0.00	2.76	0.28
3+ children	72.47	+2.73	65.19	+1.21	7.28	0.04	1.52	0.65
Birth at the year of the interview								
No birth (reference)	70.66		64.18		6.48	0.00		
Birth	73.40	+2.74	66.30	+2.12	7.1	0.00	0.62	0.51

Data are from SOEP v.34, own calculations; estimates are based on model M3. The predictions are made for age 30. All other variables are fixed at their mean for the prediction. Differences in predicted weight are calculated as weight of lower educated women minus weight of higher educated women in each of the motherhood categories. Differences in change are calculated as change in weight among lower educated women minus change in weight of higher educated women in each of the motherhood categories.

The results in Table 3 show that lower educated women were heavier than higher educated women even when childless. These differences amounted to about 6 kg at the age of 30. The effect of the initial transition into motherhood was small and slightly stronger in higher educated women, who gained approximately 0.2 kg more weight. In contrast, the effect of having two children was much stronger in lower educated women. Compared to having no children, having two children was associated with an increase of 3.22 kg among lower educated women, compared to only 0.46 kg among higher educated women. Differences in the effect of having at least three children were also substantial, as lower educated women gained about 1.5 kg more than higher educated women. Finally, the immediate effect of motherhood was associated with an increase of about 2.7 kg in lower educated women and with a slightly smaller weight gain in higher educated women (2.1 kg). This suggests a slightly larger short-term effect of gestational weight gain for lower than for higher educated women.

In the final step of the analysis, we assessed the extent to which education differences in the prevalence of motherhood and in vulnerability to its effects on weight gain accounted for diverging education gaps in body weight. Based on the estimates from model M3 and according to Eqs 4 (for the prevalence of motherhood) and 5 (for the prevalence and effects of motherhood), we calculated counterfactual body weight trajectories for lower educated women, replacing their average prevalence and effects of motherhood by the prevalence and effects of motherhood found in higher educated women.

The results are presented in Fig 4 and in Table 4. In Fig 4, unadjusted predictions in weight development for higher educated women are represented by the black solid reference line.

**Table 4:** Counterfactual analysis of change in educational difference in body weight due to differences in the prevalence and vulnerability to motherhood.

		*Prevalence*				*Prevalence and Vulnerability*			
Age	Unadjusted differences in kg	Counterfactual diff. in kg	Explained diff. in kg	Explained diff. in %	p-value	Counterfactual diff. in kg	Explained diff. in kg	Explained diff. in %	p-value
20	3.60	3.18	-0.42	-11.63	0.06	3.18	-0.42	-11.58	0.29
25	4.92	3.84*	-1.09	-22.04	0.04	3.78	-1.14	-23.21	0.25
30	6.19	5.16	-1.02	-16.56	0.19	4.79	-1.40	-22.58	0.37
35	7.40	7.13	-0.26	-3.57	0.67	6.14	-1.26	-17.01	0.42
40	8.55	8.00	-0.13	-1.47	0.82	7.24	-1.31	-15.29	0.39
45	9.64	9.43	-0.21	-2.14	0.71	8.29	-1.34	-13.95	0.37

Data are from SOEP v.34; own calculations; “Unadjusted differences” indicate estimated differences between lower and higher educated women at different ages based on model M1 for weight; “Counterfactual differences” indicate absolute size of the differences between lower and higher educated women based on model M3 under assumption that lower educated women have the same prevalence (see Table 1) and effects of motherhood (see Table 3) as higher educated women; “Explained differences in kg” = “Unadjusted difference”–“Adjusted difference”; “Explained differences in %” = $\frac{Change\;due\;to\;motherhood\;in\;kg/PP}{Unadjusted\;difference\;in\;kg/PP}$; p-values refer to the test of the hypothesis that explained differences are equal to zero.

Unadjusted predictions for lower educated women are represented by the black dashed line. The grey dashed line represents counterfactual estimates pertaining to lower educated women conditioned on the prevalence of motherhood found for higher educated women and the effects of lower educated women. The grey solid line indicates counterfactual predictions for lower educated women conditioned on the prevalence and the effects of the higher educated women. If the prevalence of motherhood fully accounted for the education gap in body weight, the grey dashed line would converge with the solid black reference line; if the prevalence of motherhood did not account for the education gap in body weight, the grey dashed line would converge with the dashed black line. The same holds for the combination of prevalence and effects (grey solid line). Table 4 shows the corresponding calculations of differences in body weight between higher and lower educated women at different ages as well as the degree of absolute (kg) and relative change (%) in these differences under our counterfactual scenarios.

As visible in Fig 4, the body weight trajectory of lower educated women moved slightly closer to the reference line of higher educated women under the counterfactual condition of the same prevalence of motherhood. The explanatory power of the prevalence of motherhood in terms of absolute body weight differences peaked between the ages of 25 and 30, when the prevalence of motherhood explained about 1 kg of differences in body weight (Table 4). After the age of 30, the explanatory power of the prevalence of motherhood declined steadily.

**Fig 5:**
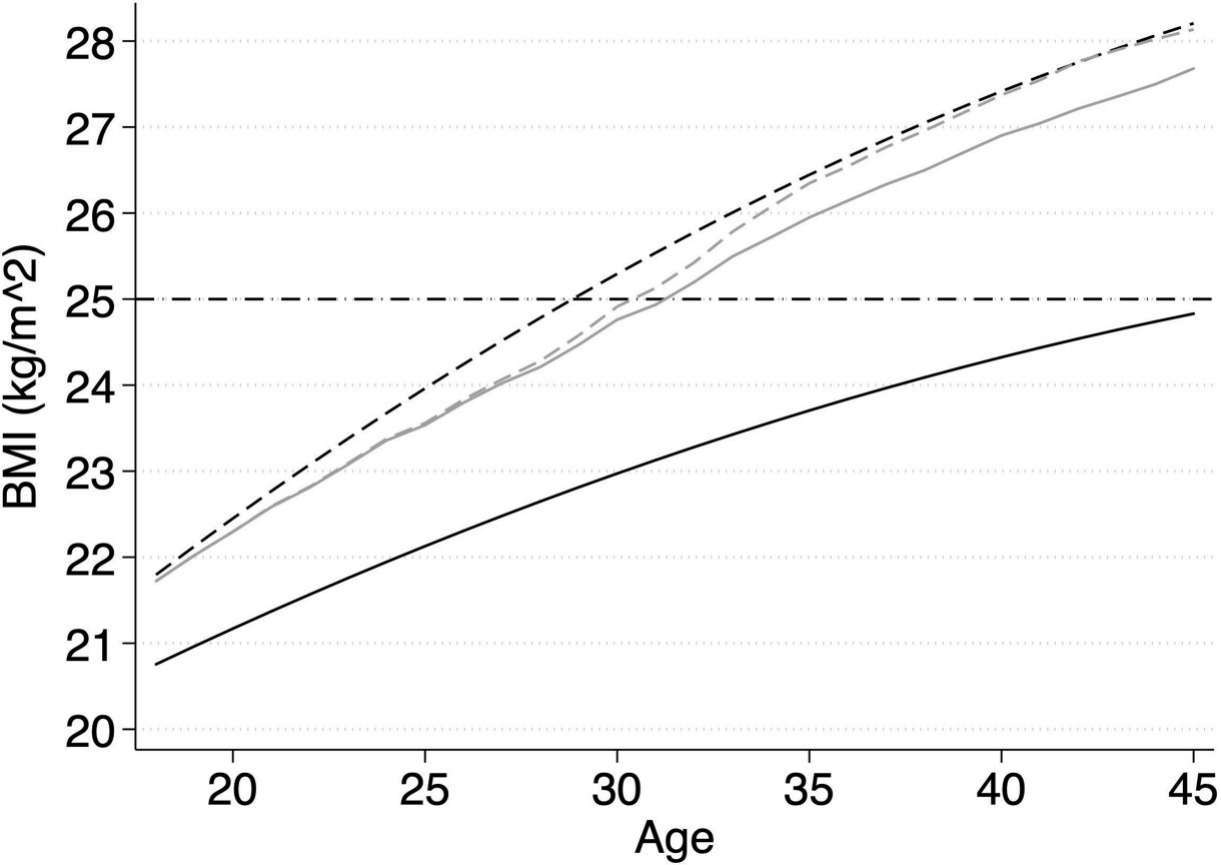
Predicted Body Mass Index (BMI) for lower and higher educated women with and without adjustment for motherhood. SOEP data, v.34; own calculations; height is fixed at the average of 167 cm in both models; black solid reference line = body weight of higher educated women, black dashed line = difference in body weight between lower educated women and higher educated women based on model M1, Table 2; grey dashed line = difference in body weight between lower educated women and higher educated women adjusted for differences in prevalence of motherhood; grey solid line = difference in body weight between lower educated women and higher educated women adjusted for differences in prevalence and effects of motherhood (M3), Table 2. The dotted horizontal line at 25 indicates the threshold to overweight according to the WHO definition.

The second counterfactual scenario, in which lower educated women are assumed to have the same prevalence and the same effects of motherhood as higher educated women, yielded similar results until the age of 25. This means that until this age, differences in the effects of motherhood on body weight did not account for the increase of education gaps in body weight. After this age, when the contribution of the isolated differences in the prevalence of motherhood steadily declined, the contribution of education differences in the effects of motherhood emerged and increased with age. At the age of 30, the differential effects of motherhood accounted for about 0.4 kg (in addition to the prevalence of motherhood). From the age of 35 onwards, differential effects of motherhood accounted for approximately 1 kg (Table 4). In absolute terms, this means that differences in vulnerability to motherhood explained about as much as differences in the prevalence of motherhood when considering the entire reproductive period. This contribution is limited but not trivial. As visible in Fig 5, in which the results are presented in terms of BMI, lower educated women would stay below the threshold to overweight (BMI > 25) about 2 years longer if they had the same prevalence and effects of motherhood as higher educated women.

Yet, a substantial part of the increase of education differences in body weight remained unexplained. Fig 4 indicates that even when differences in the prevalence and in the effects of motherhood were accounted for, education differences in body weight increased with age.

## Discussion

Previous research has found that education differences in women’s body weight increase throughout young and middle adulthood due to an accelerated weight gain among lower educated women [7,8,17,18]. Among men, these differences were found to emerge later in life and to increase less with age. Although this phenomenon is well documented, the underlying mechanisms are not well understood. In the present study, we assessed the extent to which motherhood explained this life-course pattern.

We examined the role of motherhood in terms of education differences in (a) the prevalence of motherhood and (b) the vulnerability to its effects on weight gain. This focus is an innovation in studies of education differences in body weight, taken from health research that has examined explanatory factors both in terms of risk (also called prevalence or exposure) and vulnerability (also called susceptibility or penalty) [60–62]. We conceptualized motherhood as a turning point in women’s weight trajectories, channeling lower educated women into trajectories of greater weight gain earlier in life. In the German context of our investigation, motherhood represented a potential turning point for education differences in weight trajectories, as the timing of motherhood and the number of children are strongly stratified, whereby lower educated women have more children and experience motherhood earlier in life. Moreover, education groups differ in their personal, social, and economic resources that can offer protection from the effects of motherhood on weight gain.

In line with our expectations, we found that motherhood was associated with substantial weight gain, was more prevalent among the lower educated, and differed by education in its effects on body weight. As a result, motherhood explained a part of the observed life-course increase of educational differences in body weight. The prevalence of motherhood and vulnerability to its effects on weight gain showed distinct explanatory contributions across the reproductive age range. Prevalence explained more of the gaps observed at younger ages, effects explained more of the gaps observed at older ages. These results show the importance of considering both pathways. If we had ignored education differences in the effects of motherhood, we would have erroneously concluded that motherhood barely explains education gaps in body weight after the age of 35.

These results fit with the observed education differences in the prevalence and effects of motherhood. Until the age of 35, having one or two children was more common among lower educated women than among higher educated women. Beyond this age, education differences in terms of prevalence converged, but motherhood remained influential, as lower educated women experienced stronger effects of motherhood on weight gain, particularly when having two or more children.

Overall, the explanatory power of motherhood was limited, ranging between 14% (at the age of 45) and 23% (at the age of 25) of differences in body weight observed between lower and higher educated women. Although differences in the prevalence of motherhood and vulnerability to its effects on weight gain were largely in line with our expectations, accounting for these differences still left a major share of the observed increase in educational differences in body weight unexplained.

Another interpretation is that motherhood is not the main turning point, but only one of many factors that account for the life-course increase of education differences in women’s body weight. For example, similar to motherhood, the school-to-work transition is stratified by education, occurs early in life, and is related to body weight [63]. Moreover, this transition might be associated with more weight gain among lower educated women than among lower educated men. Lower educated women more often work in routine jobs that are associated with greater weight gain, whereas lower educated men more often work in manual jobs that are unrelated to weight gain, particularly early in life [63].

Another potentially important links are biological. First, genetic studies (mainly based on twin comparisons) have suggested that body weight and its increase with age are highly heritable. A study of Finnish twins found that shared genes explained 60% of the variance in BMI and 64% of the variance in its increase [64]. According to the literature, genetic similarity accounts for approximately 30% of the relationship between education and BMI [65].

Second, education differences in the timing of menarche may partially explain why lower educated women were on a steeper weight trajectory already in their early 20s. Studies show that menarche is associates with weight gain [66] and that the onset in menarche is earlier in girls with lower socioeconomic status [67]. Moreover, girls who later receive lower education degrees start being sexually active and using hormonal contraceptives earlier [68,69]. Hormonal contraceptives, in turn, are positively associated with weight gain. Unfortunately, our data did not include the information required for considering those factors empirically.

Finally, we note that the contribution of motherhood may be underestimated due to data limitations. First, although our data covered an extensive window of observation, we cannot exclude the possibility that our measures of motherhood did not fully capture its association with body weight. In particular, we did not observe all transitions to pregnancies and higher parities during our observation window. Some women already had children before their initial observation in 2002. These women did not contribute to the calculation of the effects of motherhood. Future research could improve on our data by following a cohort of women observed before their initial transition into motherhood and across their entire reproductive period. Second, our data included only self-reported measures of body weight. Although widely considered as valid, self-reported body weight was shown to be less accurate than objectively measured body weight. Moreover, the discrepancies between self-reported and measured weight were shown to vary by age, education and sex, whereby higher educated young women tend underestimate their weight more strongly [49–51]. The size of the discrepancies between self-reported and measured body weight varies between studies, amounting to about 1,5 kg on average [52]. Because the differences we find are much larger already during the mid 20s, it is unlikely that they mainly reflect measurement error. Nevertheless, the exact sizes of our estimated effects should be interpreted with caution.

We conclude that the pattern of increasing differences in body weight between education groups is partially due to differences in the prevalence and effects of motherhood. This finding points to a potentially fruitful avenue for policies and practitioners aiming at reducing social differences in body weight and associated adverse outcomes. As mothers are usually in regular contact with health providers during pregnancies and the first years of a child’s life, advice can specifically target lower educated mothers’ awareness of preventive behaviors that can limit excessive weight gain during pregnancy and after motherhood.

## Supporting information

S1 Appendix (PDF)
