# Supplementary material for: Education differences in women’s body weight trajectories: The role of motherhood
Source: PLoS One. 2020 Sep 21;15(9):e0236487. doi: 10.1371/journal.pone.0236487 (PMC7505466; doi:10.1371/journal.pone.0236487)
Supplement: S1 Appendix — (PDF) [file pone.0236487.s001.pdf]

Online Appendix

Table A1: Sample restrictions

Sample restrictions	*Individuals*	*Observations*
Full sample in 2002	29,101	142,149
Only women	14,857	73,558
Aged 17 to 45; younger than 35 at the first wave	3,461	15,911
Born in Germany or <15 years old upon migration	3,255	15,062
BMI range between 16 and 58	3,251	15,021
No missing values on each of the analytic variables	3,052	14,283
Participation in at least two waves	2,668	13,899
Final sample size	2,668	13,899

Note: SOEP data, v.34; own calculations.

Table A2: Number of observations by education and age

Age	*Lower education*	*Intermediate education*	*Higher education*
17	31	66	26
18	20	57	36
19	64	125	49
20	55	114	69
21	91	190	75
22	63	173	93
23	90	233	116
24	78	217	122
25	94	280	135
26	82	254	153
27	105	282	144
28	98	274	171
29	118	307	166
30	118	320	181
31	151	363	173
32	142	372	193
33	162	414	191
34	174	437	199
35	179	464	210
36	152	378	176
37	158	376	162
38	124	318	131
39	128	295	130
40	99	255	106
41	102	230	117
42	68	203	87
43	74	169	88
44	48	144	55
45	37	82	48
Total	2,905	7,392	3,602

Note: SOEP data, v.34; own calculations.

Table A3: Number of observations per educational group and number of children

	Maximum number of children observed			
Education	0	1	2	3
Higher	897	670	784	554
Middle	3083	1818	1916	575
Lower	2132	640	669	161
Total	6112	3128	3369	1290

Note: SOEP data, v.34; own calculations.

Table A4: Number of observations per educational group and observed birth

	Observed births	
Education	No birth	Birth
Higher	1876	1029
Middle	4302	3090
Lower	1918	1684
Total	8096	5803

Note: SOEP data, v.34; own calculations.

a) Currently pregnant included

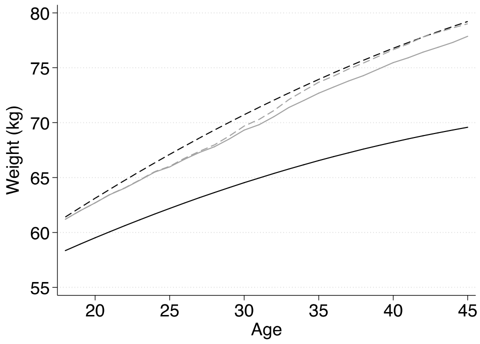

b) Currently pregnant excluded

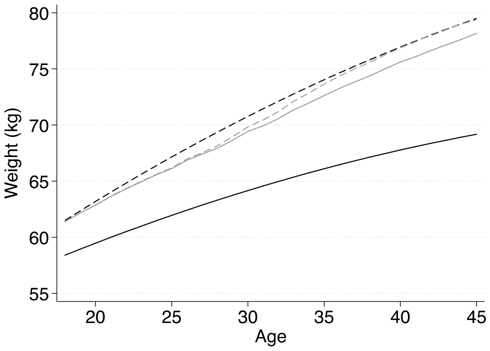

FIGURE A1: ROBUSTNESS CHECK: EXCLUSION OF OBSERVATIONS FROM CURRENTLY PREGNANT WOMEN

Note: SOEP data, v.34; own calculations. height is fixed at the average of 167 cm in both models.
black solid reference line = body weight of higher educated women, black dashed line = difference in body weight between lower educated women and higher educated women based on model M1, Table 2; grey dashed line = difference in body weight between lower educated women and higher educated women adjusted for differences in prevalence of motherhood; grey solid line = difference in body weight between lower educated women and higher educated women adjusted for differences in prevalence and effects of motherhood (M3), Table 2;
